# Decoding self-motion: insights into visual-vestibular causal inference

**DOI:** 10.3389/fnhum.2026.1879282

**Published:** 2026-09-22

**Authors:** Huizhe Sun, Aihua Chen, Fu Zeng

**Affiliations:** Key Laboratory of Brain Functional Genomics (Ministry of Education), East China Normal University, Shanghai, China

**Keywords:** Bayesian causal inference, multisensory processing, optic flow, self-motion perception, visual-vestibular integration

## Abstract

Self-motion perception relies on the integration of visual optic flow and vestibular signals, yet these cues are often noisy, unreliable, or mutually inconsistent. A central challenge is therefore not only how to integrate them, but also whether they should be integrated at all. Bayesian causal inference provides a framework for this problem by proposing that the brain infers whether visual and vestibular cues arise from a common cause and adjusts their integration accordingly. Behavioral findings, including disparity-dependent perceptual biases, reliability-dependent cue reweighting, and experience-dependent recalibration, are compatible with this framework, but are not diagnostic of causal inference in isolation and may also arise from alternative integration or decision mechanisms. At the neural level, studies in humans and nonhuman primates have identified a distributed self-motion network involving MSTd, VIP, VPS/PIVC, and frontal and parietal association areas. However, current evidence mainly concerns multisensory convergence, cue weighting, and conflict sensitivity rather than a direct neural representation of causal structure. Here, we review visual-vestibular causal inference from three linked perspectives: the computational framework and its domain-specific assumptions, the strength and limitations of the behavioral model evidence, and the candidate neural substrates and circuit mechanisms. We conclude by highlighting major open questions and future directions for understanding causal inference during naturalistic self-motion.

## 1 Introduction

The brain continuously combines signals from multiple sensory systems to construct a coherent representation of the body and the external world (Wallace et al., 2004; Stein et al., 2014). Classical theories of multisensory integration emphasize statistically optimal cue combination, whereby sensory signals are weighted according to their reliability to improve perceptual precision (van Beers et al., 1996; Ernst and Banks, 2002; Alais and Burr, 2004; Fetsch et al., 2009; Prsa et al., 2012). These models generally assume, either explicitly or implicitly, that the signals refer to the same underlying event. In natural environments, however, sensory inputs may be noisy, ambiguous, or generated by different sources. Perception therefore depends not only on how cues are weighted, but also on whether they should be combined. Bayesian causal inference (BCI) addresses this problem by estimating whether sensory signals arise from a common or separate source and using this inferred causal relationship to determine how signals should be combined to form a perceptual estimate (Kording et al., 2007; Shams and Beierholm, 2010; Kayser and Shams, 2015; Shams and Beierholm, 2022).

Visual-vestibular self-motion perception provides a particularly informative setting for studying this problem. During movement through space, optic flow and vestibular signals provide complementary information about heading and motion (Gu et al., 2008; Fetsch et al., 2009; Laurens and Angelaki, 2017; Acerbi et al., 2018), but their physical origins are not equivalent. Vestibular signals primarily reflect inertial motion of the head (Merfeld et al., 1999; Angelaki, 2004), whereas retinal image motion may arise from observer motion, movement of objects or the visual environment, or a combination of these events (Gibson, 1950; Warren and Hannon, 1988). A visual-vestibular discrepancy may therefore reflect sensory uncertainty, but it may also indicate that part of the visual motion arises from an external source. This ambiguity is evident when movement of a nearby train or visual surround induces a compelling sense of self-motion while inertial signals indicate that the observer remains stationary; conversely, during observer motion through a stationary environment, optic flow and vestibular input may provide complementary measurements of the same self-motion event (Gu et al., 2008; Fetsch et al., 2009).

Many influential heading experiments have examined this problem using motion platforms that impose passive translation while optic flow is manipulated independently (Gu et al., 2008; Fetsch et al., 2009; Acerbi et al., 2018). These paradigms provide precise control over visual and inertial cues, but do not include the motor commands, efference copy, and much of the proprioceptive information available during active motion. Accordingly, the visual-vestibular causal-inference models reviewed here are formulated for passive self-motion and primarily address whether visual and vestibular measurements should be attributed to the same observer-motion event or to separate sources. Extending this framework to active and naturalistic movement remains an important challenge (Acerbi et al., 2018).

Behavioral studies have identified several forms of flexible visual-vestibular interaction. When the reliability of the two cues differs, observers tend to place greater weight on the more reliable cue, a pattern known as reliability-dependent cue weighting (Fetsch et al., 2009). When the cues provide discrepant motion signals, the perceptual estimate can be biased toward one cue as the visual-vestibular disparity changes, producing a disparity-dependent perceptual bias (Rodriguez and Crane, 2019). Repeated exposure to discrepant cues can also alter subsequent perceptual estimates, reflecting experience-dependent recalibration (Zaidel et al., 2011; Zaidel et al., 2013). These findings demonstrate that visual and vestibular information is combined flexibly, but they are not, by themselves, direct evidence for causal inference. Reliability-dependent reweighting is also predicted by optimal cue-integration models that assume a common cause. Disparity-dependent perceptual biases under cue conflict may arise from nonlinear or robust cue combination, bounded fusion, or trial-to-trial variability in whether the cues are integrated or segregated. Recalibration may reflect changes in unisensory estimates or decision processes rather than an explicit inference about the causal relationship between cues. Stronger evidence comes from experiments that examine broad ranges of disparity, manipulate the likely source of visual motion, or compare causal-inference models with competing accounts of multisensory integration and decision strategy (de Winkel et al., 2017; Acerbi et al., 2018; Dokka et al., 2019). These studies indicate that observers take the likely relationship between the cues into account, although they do not establish a unique Bayesian decision strategy.

At the neural level, visual and vestibular signals converge in a distributed self-motion network that includes the dorsal medial superior temporal area (MSTd), ventral intraparietal area (VIP), visual posterior sylvian area (VPS) and parieto-insular vestibular cortex (PIVC), as well as frontal and parietal association regions (Gu et al., 2008; Chen et al., 2010, 2011a,b,c; Fetsch et al., 2011). Studies in parts of this network have shown that neural responses in these areas vary with cue reliability, congruence, and disparity, providing possible mechanisms for reliability-dependent cue weighting, conflict detection, and flexible multisensory integration. However, these response modulations do not by themselves show that neural activity represents whether visual and vestibular cues are inferred to arise from a common or separate source. Studies in other multisensory domains have reported hierarchical and population-level neural signatures related to causal judgments or model-derived estimates of common-cause probability (Rohe and Noppeney, 2015; Aller and Noppeney, 2019; Cao et al., 2019; Rohe et al., 2019; Qi et al., 2022). Comparable evidence in visual-vestibular self-motion perception remains limited, leaving open whether neural activity in the self-motion network reflects causal inference itself or the component computations, such as sensory reliability estimation, cue weighting, conflict detection, and perceptual decision-making, that may support it.

Previous reviews have approached these issues from two complementary perspectives. Broad accounts of Bayesian causal inference have treated source inference as a general principle of multisensory perception (Shams and Beierholm, 2022), whereas reviews of vestibular and self-motion processing have focused mainly on sensory transformations, reliability-weighted cue combination, internal models, and distributed neural representations (Angelaki and Cullen, 2008; Laurens and Angelaki, 2017). Recent methodological work has further emphasized that common measures of multisensory interaction do not, on their own, uniquely distinguish sensory integration from causal inference (Zhu et al., 2026). What remains unclear is how strongly causal inference has been established specifically for visual-vestibular self-motion, and which behavioral and neural findings provide evidence for causal inference beyond flexible cue weighting and multisensory interaction.

The present review addresses this gap by integrating computational, behavioral, and neural perspectives. We examine how causal hypotheses are formulated for visual and vestibular signals and evaluate the evidence for causal inference at both behavioral and neural levels. In particular, we distinguish flexible cue weighting and disparity-dependent perceptual biases from stronger behavioral evidence based on source attribution and model comparison, and consider whether current neural findings reveal representations of causal structure or instead reflect the component processes that may support such inference. Finally, we discuss challenges that are particularly relevant to self-motion, including the ambiguous relationship between optic flow and physical movement and the limits of passive laboratory paradigms, and highlight how future experiments could more directly test causal inference during active and naturalistic movement.

## 2 Conceptual framework of causal inference

Bayesian causal inference extends conventional cue-integration models by allowing the observer to consider whether sensory signals should be attributed to the same event. It is not a single integration rule, but a class of observer models that combines assumptions about how sensory observations are generated, how likely different sources are before the current observations are received, how self-motion is estimated under each causal hypothesis, and how these estimates are translated into a behavioral response (Kording et al., 2007; Shams and Beierholm, 2010; Shams and Beierholm, 2022).

### 2.1 What is special about visual-vestibular causal inference?

Visual and vestibular signals differ from many other multisensory pairs because they are not always redundant measurements of the same physical event. Vestibular signals reflect head rotation and linear acceleration relative to gravity, whereas retinal motion may arise from observer motion, moving objects, the surrounding environment, eye movements, or combinations of these events. A visual-vestibular discrepancy may therefore reflect sensory uncertainty, but it may also indicate that the two signals have different physical origins (Gibson, 1950; Merfeld et al., 1999; Angelaki, 2004; Laurens and Angelaki, 2017).

This causal problem also changes between passive and active movement. Many laboratory studies independently manipulate optic flow and inertial motion during passive platform movement (Gu et al., 2008; Fetsch et al., 2009; de Winkel et al., 2017; Acerbi et al., 2018), whereas natural self-motion additionally involves proprioception, motor commands, and efference copy. Virtual-reality and simulator environments create further conflicts because visual motion can indicate self-motion without corresponding inertial input. The common- versus separate-cause model therefore provides a useful simplification, but it may not capture the dynamic and multi-source causal structures present during natural self-motion.

### 2.2 A domain-specific common- versus separate-cause model

Despite this complexity, most controlled visual-vestibular experiments can be described using a common-versus-separate cause model. The model does not represent every possible physical source of visual and vestibular signals. Instead, it asks whether the two sensory measurements can be explained by the same self-motion event or should be treated as arising from separate motion states.

In these paradigms, the observer receives a visual measurement, $x_{\mathrm{vis}}$ , and a vestibular measurement, $x_{\text{vest}}$ . Under the common-cause hypothesis ( C=1 ), both measurements are generated by a shared self-motion state, s , such as heading direction:

$$p(x_{\mathrm{vis}},x_{\text{vest}}|s,C=1)=p(x_{\mathrm{vis}}|s)p(x_{\text{vest}}|s)$$

Under the separate-causes hypothesis ( C=2 ), visual and vestibular measurements are associated with distinct motion states:

$$p(x_{\mathrm{vis}},x_{\text{vest}}|s_{\mathrm{vis}},s_{\text{vest}},C=2)=p(x_{\mathrm{vis}}|s_{\mathrm{vis}})p(x_{\text{vest}}|s_{\text{vest}})$$

Here, C=1 and C=2 represent task-specific hypotheses about whether the two sensory measurements can be explained by the same self-motion state. Under C=1 , both cues are linked to a shared motion state. Under C=2 , they are assigned to distinct latent motion states. The vestibular state, $s_{\text{vest}}$ represents inertial self-motion, whereas the visual state, $s_{\mathrm{vis}}$ represents the motion specified by visual input and may reflect observer motion, external object or scene motion, or a combination of these components. The two hypotheses therefore differ in how the visual and vestibular measurements are related to the underlying motion states.

The sensory likelihoods describe the uncertainty with which each modality measures its underlying state and are often approximated by Gaussian distributions:

$$x_{m}\sim N(s_{m},\sigma_{m}^{2})$$

where $\sigma_{m}^{2}$ denotes modality-specific sensory variance. Narrower likelihoods indicate more reliable measurements.

The model also includes two distinct priors. The causal prior, P(C=1) = $p_{\text{common}}$ , describes the prior probability that visual and vestibular signals arise from the same event. The motion-state prior, p(s) , describes prior expectations about heading or motion direction. The former acts on the causal hypotheses, whereas the latter constrains the estimated motion state and may also influence the evidence for each hypothesis.

This common- versus separate-cause model provides a useful approximation for passive heading paradigms, but it is not a complete representation of visual-vestibular causality in natural behavior. Active movement additionally involves motor commands, proprioception, and efference copy, while visual and vestibular signals also differ in their temporal dynamics. Modeling active or dynamically changing self-motion may therefore require additional sources of information and more complex causal relationships. Active and passive movement should consequently be regarded as experimental conditions rather than the two causal hypotheses themselves.

### 2.3 Inferring causal structure and estimating self-motion

Given the visual and vestibular measurements, the observer evaluates how well they are explained by the common-cause and separate-causes hypotheses (Kording et al., 2007). The posterior probability of a common cause is given by:

$$\begin{array}{l} P(C=1|x_{\mathrm{vis}},x_{\text{vest}})=\\ \frac{p(x_{\mathrm{vis}},x_{\text{vest}}|\;C=1)P(C=1)}{\left(x_{\mathrm{vis}},x_{\text{vest}}|\;C=1)P(C=1)+(x_{\mathrm{vis}},x_{\text{vest}}|\;C=2)P(C=2\right)} \end{array}$$

This probability depends jointly on the sensory evidence and the prior probability of a common cause. Similar visual and vestibular measurements generally provide stronger evidence for a common cause, whereas larger discrepancies favor separate causes. The effect of disparity also depends on sensory uncertainty: a given mismatch provides stronger evidence against a common source when both measurements are precise than when one or both are noisy (Kording et al., 2007; Shams and Beierholm, 2010; Acerbi et al., 2018). Sensory reliability therefore influences both the relative weighting of the cues and the evidence used to compare the causal hypotheses.

The model also estimates self-motion conditional on each causal hypothesis. Under C=1 , visual and vestibular signals contribute to a shared estimate of self-motion:

$$p(s|x_{\mathrm{vis}},x_{\text{vest}},C=1)\propto p(x_{\mathrm{vis}}|s)p(x_{\text{vest}}|s)\;p(s)$$

Under C=2 , separate visual and vestibular states are estimated:

$$\begin{array}{l} p(s_{\mathrm{vis}},s_{\text{vest}}|x_{\mathrm{vis}},x_{\text{vest}},C=2)\propto p(x_{\mathrm{vis}}|s_{\mathrm{vis}})\\ p(x_{\text{vest}}|s_{\text{vest}})\;p(s_{\mathrm{vis}})p(s_{\text{vest}}) \end{array}$$

Causal inference and motion-state estimation are therefore defined within the same generative model: causal inference determines how strongly the cues should contribute to a shared versus separate representation, whereas the sensory likelihoods and motion-state priors determine the estimated state under each hypothesis. These posterior probabilities and state estimates describe the observer’s internal inference, but they do not by themselves specify the behavioral response.

### 2.4 Task-dependent readout and decision strategies

The posterior probabilities of the causal hypotheses and the state estimates derived under each hypothesis must ultimately be translated into a task-specific response. In an inertial-heading task, the reported variable is the perceived direction of body motion. When the cues are treated as arising from a common cause, the report is influenced by the integrated visual-vestibular estimate. When they are treated separately, the vestibular or inertial estimate is the task-relevant quantity. For a visual-heading task, the visual estimate would instead be task relevant. Thus, causal inference specifies beliefs about the origins of the sensory signals, but the behavioral response also depends on the task and the decision rule.

Several decision or readout strategies can map the inferred states onto behavior. Under model averaging, the estimates derived under the two causal hypotheses are weighted by their posterior probabilities (Rohe and Noppeney, 2015). For an inertial-heading report:

$${\hat{s}}_{\text{inertial}}=P(C=1|x_{\mathrm{vis}},x_{\text{vest}}){\hat{s}}_{C=1}+P(C=2|,x_{\mathrm{vis}}|,x_{\text{vest}}){\hat{s}}_{\text{vest},C=2}$$

Model averaging can therefore produce graded or partial cue attraction when the evidence for a common source is intermediate. Model selection instead uses the estimate associated with the more probable causal hypothesis. Probability matching selects between the hypotheses in proportion to their posterior probabilities, whereas criterion-based strategies integrate or separate the cues depending on whether disparity or another decision variable crosses a threshold. These are not alternative causal hypotheses; they are different ways of converting uncertainty about causal structure into a behavioral response.

This distinction is important because the same underlying causal inference can produce different behavioral patterns depending on the readout rule. Explicit unity judgments concern whether the cues are perceived as sharing a source, whereas implicit heading judgments reflect a motion estimate shaped by sensory evidence, source attribution, and the decision rule. The same sensory observations may therefore produce different responses across explicit and implicit tasks.

Additional assumptions are needed to describe how internal estimates become observed responses. The loss function specifies how an internal posterior estimate is converted into an action; for example, squared-error loss favors the posterior mean (Kording and Wolpert, 2004), whereas categorical or criterion-based tasks may use different decision rules (Acerbi et al., 2018). Response noise captures variability introduced during decision formation or response execution, whereas lapse parameters capture occasional stimulus-independent errors, such as failures of attention or random responses. These sources of response variability should be distinguished from sensory uncertainty, which affects the precision of the sensory measurements earlier in the inference process. Model comparisons should therefore specify the assumed loss function, response-noise, and lapse processes and whether their parameters are fixed or shared across tasks.

Finally, the quantities involved in inference and behavioral readout may themselves change with experience. Standard implementations typically treat the common-cause prior, sensory uncertainty, and response mapping as fixed within an experiment. Repeated exposure to particular visual-vestibular relationships may instead alter the prior probability of a common source, recalibrate sensory estimates or cross-modal mappings, change sensory uncertainty, or shift response criteria. Thus, history-dependent changes in behavior do not uniquely imply an update of the common-cause prior; they may arise from changes at multiple stages of the inference-to-response cascade. This distinction is particularly important when interpreting experience-dependent recalibration as evidence for causal learning. Figure 1 summarizes the overall computational sequence from sensory observations to causal inference, state estimation, and behavioral readout, whereas Table 1 lists the principal model components, their roles, and the quantities that may be fixed or fitted across studies.

**Figure 1:**
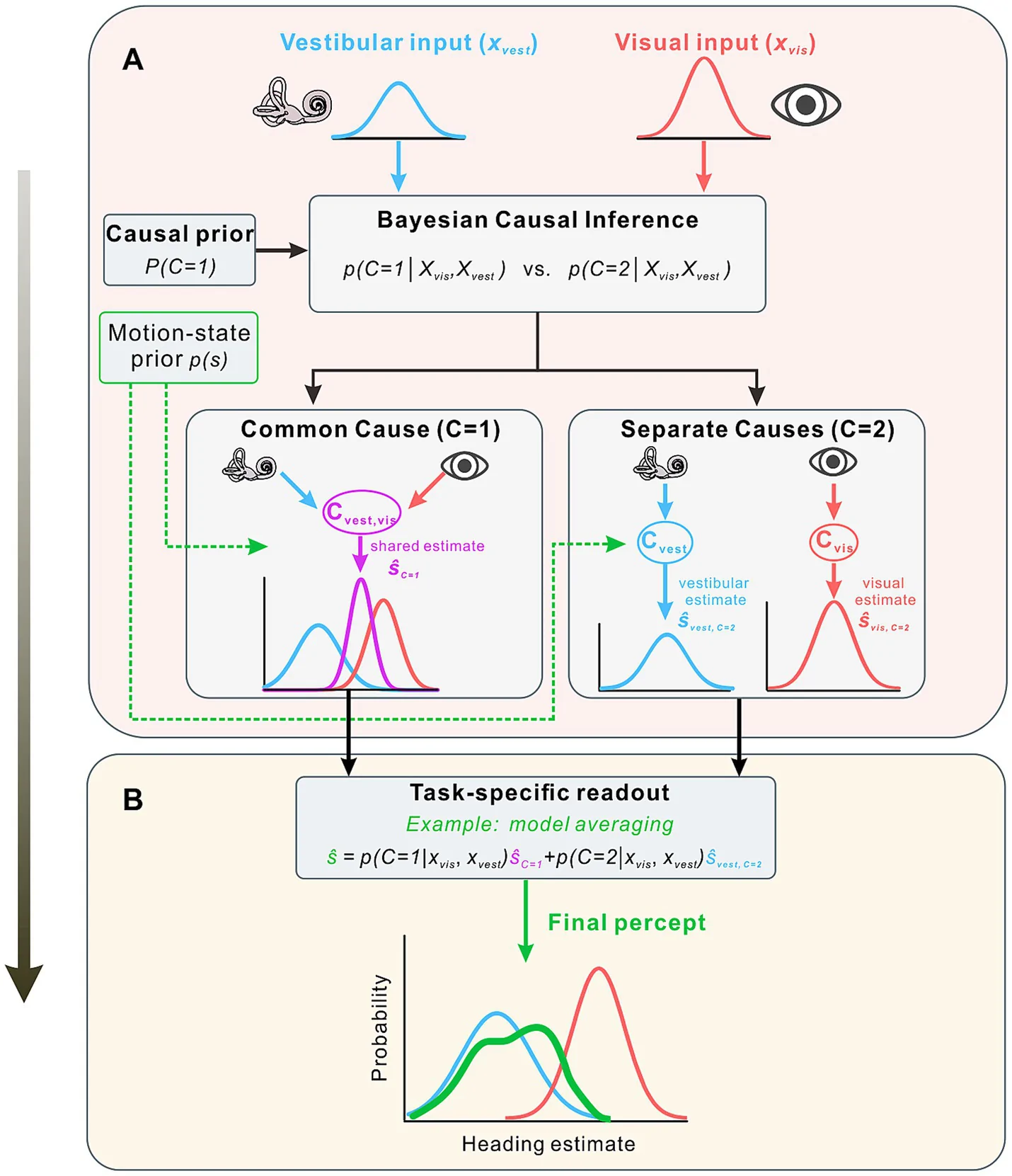
Simplified computational framework for visual-vestibular Bayesian causal inference in self-motion perception. **(A)** Visual ( $x_{\mathrm{vis}}$ ) and vestibular ( $x_{\text{vest}}$ ) measurements are evaluated under two causal hypotheses. The causal prior, *p*(*C* = 1), influences the inference of whether the cues arise from a common source (*C* = 1) or from separate causes (*C* = 2). The motion-state prior, p(s) , contributes to the estimation of heading or self-motion state under each hypothesis. Under the common-cause hypothesis, visual and vestibular cues are combined to form a shared self-motion estimate. Under the separate-causes hypothesis, modality-specific estimates are computed independently. **(B)** The posterior probabilities of the causal hypotheses and the corresponding state estimates are translated into a task-specific response. Model averaging is shown here as one example, in which estimates under different causal hypotheses are weighted by their posterior probabilities. Other possible readout strategies include model selection, probability matching, and criterion-based rules. The final report therefore depends not only on sensory evidence and priors, but also on the task-specific mapping from internal estimates to behavior.

**Table 1:** Core components of visual-vestibular Bayesian causal-inference models.

Model component	Role	Typical assumptions or parameters
Sensory likelihoods	Describe visual and vestibular measurements and their uncertainty	Modality-specific bias and variance; likelihood form
Motion-state prior	Represents prior expectations about heading or motion	Prior mean and width
Causal prior	Represents the prior probability of a common source	*p*_common_ = *p*(*C* = 1)
Causal hypotheses	Specify whether the measurements arise from shared or separate latent states	*C* = 1: shared state; *C* = 2: separate visual and vestibular states
State estimates	Provide estimates conditional on each causal hypothesis	Integrated estimate under *C* = 1; visual and vestibular estimates under *C* = 2
Task-specific readout	Determines which estimate is reported and how causal uncertainty affects the response	Inertial-heading, visual-heading, or unity judgment; model averaging, selection, probability matching, or criterion rule
Loss and response model	Converts the internal estimate into an observed response	Loss function, decision or response noise
Lapse process	Accounts for stimulus-independent errors	Lapse probability

The precise parameterization varies across studies. Some quantities are estimated from unisensory data, whereas others are fitted jointly, and not all models include separate response-noise or lapse parameters.

### 2.5 Model comparison and limits of inference

Quantitative model comparison is important for evaluating causal-inference accounts, but its interpretation depends on which models are included in the comparison (Table 2). A causal-inference model that outperforms forced fusion shows that obligatory integration cannot explain the tested behavior. It does not uniquely establish Bayesian causal inference if other plausible accounts, such as nonlinear or robust cue combination, criterion-based switching, probability matching, or mixtures of integration and segregation, have not been considered (Knill, 2007; Wozny et al., 2010; de Winkel et al., 2017; Acerbi et al., 2018). Model evidence is therefore always relative to the alternatives that were tested.

**Table 2:** Alternative accounts of graded multisensory integration.

Model or account	How graded responses arise	Evidence needed to distinguish it	References
Forced fusion with noise	Cues are always integrated; noise produces smooth responses	Evidence for segregation or source-dependent integration	de Winkel et al. (2015); Acerbi et al. (2018)
Cue switching or model selection	The observer selects integration or segregation; variability produces graded averages	Trial-level and individual-level analyses	Kording et al. (2007); de Winkel et al. (2017); Acerbi et al. (2018)
Robust or nonlinear integration	Discrepant cues are progressively down weighted	Source effects beyond physical disparity	Knill (2007)
Probability matching or response mixture	Different estimates or strategies occur across trials	Trial-level response distributions	Wozny et al. (2010); de Winkel et al. (2017); Acerbi et al. (2018)
Bayesian causal inference	Integration depends on the inferred probability of a common source	Joint effects of disparity, reliability, task, and source attribution	Kording et al. (2007); de Winkel et al. (2017); Acerbi et al. (2018); Dokka et al. (2019)

A further difficulty is that different models or parameter combinations can produce similar behavioral predictions. Greater tolerance of visual-vestibular disparity, for example, may reflect either a stronger prior belief in a common source or greater sensory uncertainty. Similarly, graded mean responses may arise from model averaging, stochastic selection between causal hypotheses, or variability across trials and observers (Wozny et al., 2010; de Winkel et al., 2017). These alternatives are especially difficult to distinguish when analyses are based only on condition-averaged perceptual biases.

Combining multiple behavioral measures can place stronger constraints on the model. Unisensory conditions provide estimates of modality-specific bias and uncertainty, implicit heading judgments constrain the task-relevant multisensory estimate, and explicit unity judgments provide additional information about source attribution. Joint analysis of these tasks has provided stronger evidence against obligatory fusion, while also showing that fixed-criterion and fully Bayesian decision rules may remain difficult to distinguish and that some prior parameters are only weakly constrained (Acerbi et al., 2018).

Model-recovery analysis tests whether data generated by one candidate model can be correctly identified when all competing models are fitted to those data. Parameter-recovery analyses similarly assess whether known generating parameters can be recovered with acceptable accuracy (Wilson and Collins, 2019). Criteria such as AIC or BIC account for model complexity when comparing fit quality, whereas out-of-sample prediction tests whether a fitted model generalizes to data that were not used for fitting. These approaches complement one another, but none can compensate for omitted alternatives or for candidate models that make nearly indistinguishable predictions. Evidence for visual-vestibular causal inference is therefore most informative when the experimental manipulation produces separable predictions for sensory uncertainty, source attribution, and decision strategy, and when these predictions are examined at the individual and trial levels.

## 3 Behavioral evidence for visual-vestibular causal inference

Behavioral studies show that visual and vestibular signals are combined flexibly during self-motion perception (Fetsch et al., 2009; Acerbi et al., 2018; Dokka et al., 2019).

Their relative influence varies with sensory reliability, cue disparity, task demands, and previous experience. These findings rule out simple accounts based on fixed modality weights or obligatory fusion, but they do not all provide equivalent evidence for causal inference. Reliability-dependent weighting primarily describes how cues are combined, whereas studies that manipulate cue disparity, the likely source of visual motion, or explicit judgments about whether the cues share a source provide stronger tests of causal inference. Experience-dependent recalibration further demonstrates that visual-vestibular relationships are learned over time (Zaidel et al., 2011; Zaidel et al., 2013), although the underlying changes may occur at sensory, causal, or decision stages.

### 3.1 Cue conflict, integration-segregation, and source attribution

The influence of one sensory cue on another generally decreases as visual-vestibular disparity increases (Kording et al., 2007; Shams and Beierholm, 2010). At small heading disparities, perceived self-motion is often biased toward a reliability-weighted combination of the visual and vestibular estimates. As disparity increases, cross-modal attraction tends to weaken, although the magnitude and range of integration vary across tasks and observers (de Winkel et al., 2015, 2017; Acerbi et al., 2018; Rodriguez and Crane, 2019).

This graded relationship is consistent with Bayesian causal inference: increasing disparity generally reduces the probability that the two measurements arose from the same event. However, graded cue attraction is not diagnostic of model averaging or Bayesian inference. Sensory variability can smooth the behavior produced by categorical selection or threshold-based strategies, while nonlinear weighting, robust integration, and trial-to-trial mixtures of integration and segregation can produce similar mean response functions (Wozny et al., 2010; de Winkel et al., 2017; Acerbi et al., 2018). Thus, graded attraction shows that cue influence changes with disparity, but does not by itself identify the computation responsible for this change.

Early visual-vestibular studies primarily examined cue combination over relatively modest heading conflicts (Gu et al., 2008; Fetsch et al., 2009). Fetsch et al. (2009), for example, showed that observers adjusted visual and vestibular weights according to their relative reliability, providing strong evidence for flexible multisensory integration when the cues could plausibly describe the same self-motion event. Studies using broader disparity ranges subsequently found that causal-inference models could account for the reduction in visual-vestibular interaction as conflict increased (de Winkel et al., 2017; Acerbi et al., 2018). At the group level, model averaging often captured the gradual change in perceptual bias, although probability matching, in which observers stochastically select between causal hypotheses according to their posterior probabilities, segregation, or other strategies described some individuals better, indicating substantial variation in how causal uncertainty is translated into a response.

Manipulating the likely origin of visual motion provides a more direct test of causal inference than varying disparity alone. Visual motion may be interpreted as a consequence of observer movement through a stationary environment or as containing an additional contribution from object motion. Heading estimates change accordingly: visual information has a stronger influence when it is consistent with observer motion and a weaker influence when independent object motion is more plausible (Dokka et al., 2019). This result links cue integration to the inferred source of visual motion rather than to disparity alone.

The apparent boundary between integration and segregation also depends on how causal structure is probed: implicit heading judgments measure how one cue influences the reported self-motion estimate, whereas explicit unity or source judgments ask whether the cues are perceived as arising from the same event. Visual-vestibular interactions have been reported even at relatively large disparities, whereas other experiments show a clearer decline in visual influence with conflict (de Winkel et al., 2015, 2017; Rodriguez and Crane, 2019). These differences suggest that disparity is evaluated together with cue reliability, temporal correspondence, stimulus interpretation, and the reported variable. Evidence for causal inference is therefore strongest when changes in cue interaction follow manipulations of source attribution and are better explained by causal-inference models than by plausible alternatives.

### 3.2 Sensory reliability and causal inference

Visual and vestibular cues are weighted according to their sensory reliability during heading perception (Fetsch et al., 2009; Butler et al., 2010). Reducing the reliability of optic flow increases the influence of vestibular information, whereas more reliable visual motion shifts the estimate toward the visual heading (Fetsch et al., 2009). Corresponding changes in neural and behavioral cue weights have also been reported in macaques performing multisensory heading judgments (Fetsch et al., 2009; Fetsch et al., 2011). Together, these findings provide clear evidence that visual-vestibular integration is not governed by fixed modality weights.

Reliability has a broader role in causal-inference models. When visual and vestibular signals are treated as measurements of a common event, their relative reliability determines their contribution to the integrated estimate (Fetsch et al., 2009; Fetsch et al., 2010). Sensory uncertainty also affects the interpretation of disparity: a mismatch between reliable signals provides stronger evidence against a common cause than the same mismatch between noisy signals (de Winkel et al., 2017; Acerbi et al., 2018). Thus, integration may persist over a broader range of disparities when sensory uncertainty is greater.

Importantly, reliability-dependent weighting alone does not demonstrate that the observer has inferred a common or separate source, because the same weighting is predicted by optimal cue integration under an assumed common cause. Reliability becomes particularly relevant to causal inference when sensory uncertainty changes how cue disparity affects the tendency to integrate or separate the signals. Existing studies show that both reliability and disparity influence visual-vestibular behavior (Fetsch et al., 2009; de Winkel et al., 2017; Acerbi et al., 2018; Ramkhalawansingh et al., 2018), but relatively few have independently and systematically manipulated both factors within the same experimental design. Designs that separate these effects, particularly when combined with source judgments or quantitative model comparison, provide a stronger test of causal inference than reliability-dependent weighting alone.

### 3.3 Experience-dependent recalibration

Visual-vestibular estimates are also shaped by previous multisensory experience. Prolonged exposure to discrepant cues can shift subsequent unisensory and multisensory judgments, indicating that the mapping between visual and vestibular estimates can be recalibrated rather than being fixed (Zaidel et al., 2011). Such recalibration was not determined simply by the reliability of an individual cue, suggesting that adaptation reflects the relationship between the signals during the preceding multisensory experience.

Feedback provides another route for updating self-motion estimates. When participants received information about the correct heading, subsequent recalibration followed the preceding multisensory percept rather than either unisensory estimate alone (Zaidel et al., 2013). The integrated percept can therefore provide the reference against which later visual and vestibular estimates are adjusted.

These forms of learning are relevant to causal inference because previous cue relationships may influence how new visual and vestibular signals are interpreted. Repeated congruent or discrepant stimulation could alter expectations about whether the cues arise from a common source, but recalibration does not necessarily imply an update of the common-cause prior. Behavioral changes after visual-vestibular conflict may instead reflect shifts in unisensory bias, sensory uncertainty, cross-modal mappings, or response criteria (Zaidel et al., 2011; Zaidel et al., 2013; Shams and Beierholm, 2022). The visual-vestibular recalibration studies reviewed above therefore demonstrate history-dependent adaptation, but do not identify which component of the causal-inference framework is modified by experience. Distinguishing these possibilities requires experiments that separately constrain sensory recalibration, sensory uncertainty, expectations about whether the cues share a source, and decision criteria.

### 3.4 Explicit and implicit measures of causal inference

Causal inference can be assessed through explicit judgments about the relationship between sensory signals or inferred indirectly from their influence on a perceptual estimate (Figure 2). In explicit tasks, observers report whether visual and vestibular cues appear to arise from the same event (Acerbi et al., 2018). In implicit tasks, they report a task-relevant variable such as inertial heading, and causal inference is inferred from how the influence of one cue changes with disparity or source attribution (de Winkel et al., 2017; Acerbi et al., 2018; Dokka et al., 2019). These tasks measure related but distinct aspects of behavior: explicit judgments more directly assess whether the cues are perceived as sharing a source, whereas implicit judgments reflect the combined effects of sensory estimation, source attribution, and the decision rule used to generate the report.

**Figure 2:**
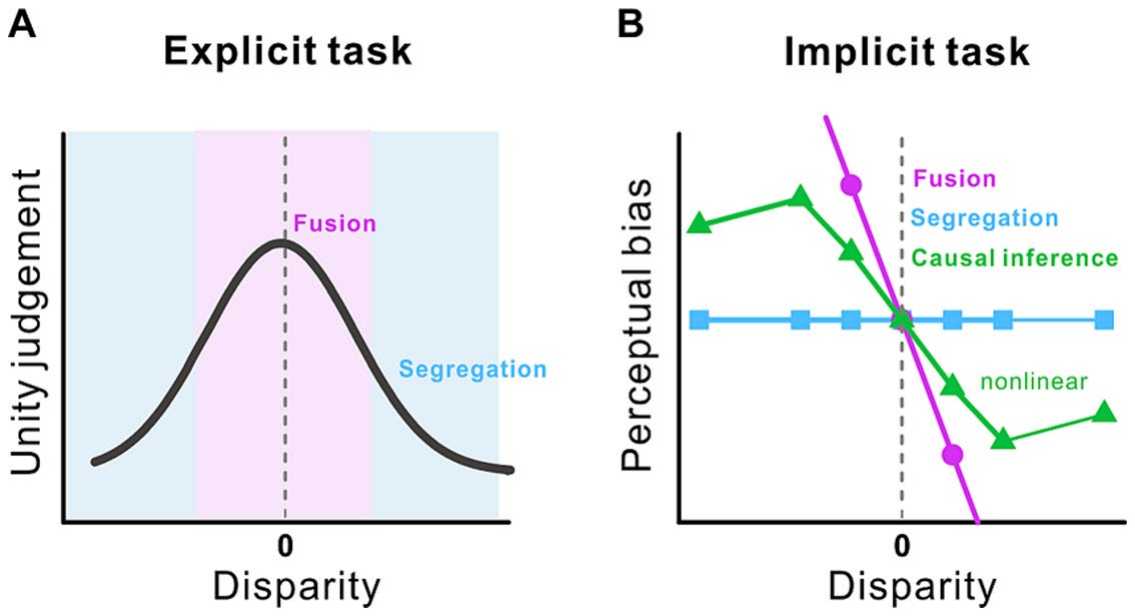
Explicit and implicit measures of visual-vestibular causal inference. **(A)** Explicit tasks require observers to judge whether visual and vestibular cues arise from the same event. **(B)** Implicit tasks measure a task-relevant percept, such as inertial heading, without requiring a direct source judgment. Cue interactions often weaken as disparity increases, but graded average responses are not unique to Bayesian causal inference and may also arise from alternative integration or decision strategies. Joint analysis of explicit and implicit measures provides stronger constraints on causal inference than either task alone.

Comparing the two types of judgment within the same visual-vestibular paradigm provides stronger constraints than either task alone. When explicit unity judgments and implicit inertial-heading estimates were examined using the same heading stimuli, both responses varied with visual-vestibular disparity, but the implicit task alone did not clearly distinguish causal-inference strategies from simpler integration accounts. Joint analysis provided stronger evidence against forced fusion and better constrained how causal inference contributed to the two tasks (Acerbi et al., 2018).

The joint results did not, however, identify a single decision strategy (Acerbi et al., 2018). A fixed criterion for selecting between integration and segregation could account for the combined explicit and implicit data at least as well as a fully Bayesian decision rule. Explicit and implicit judgments therefore should not be treated as interchangeable measures of an underlying common-cause probability, nor does agreement between them establish model averaging as a universal readout. Their main value lies in providing complementary constraints on how sensory evidence is transformed into causal judgments and self-motion estimates.

Individual differences add a further complication (de Winkel et al., 2017; Acerbi et al., 2018). Aggregate behavior may favor a graded decision rule even when individual observers are better described by model selection, probability matching, or segregation strategies (Wozny et al., 2010; de Winkel et al., 2017; Acerbi et al., 2018). Analyses of individual observers and, where possible, trial-level response distributions are therefore important for determining whether a group-level transition reflects uncertainty within observers or variation between them.

Overall, the behavioral literature provides strong evidence that visual-vestibular integration is flexible and depends on reliability, disparity, source interpretation, and previous experience (Table 3). The more specific case for causal inference rests on experiments that test a broad range of conflicts, manipulate the likely source of visual motion, and compare source-dependent models with credible alternatives. These studies indicate that observers take the likely relationship between visual and vestibular signals into account, but the decision strategy through which this information shapes perceptual reports varies across tasks and individuals.

**Table 3:** Behavioral evidence relevant to Bayesian causal inference in visual-vestibular self-motion.

Evidence category	Main finding	Relevance to BCI	References
Reliability-weighted integration	Visual and vestibular weights vary systematically with cue reliability	Provides strong evidence for flexible multisensory integration, but does not by itself demonstrate causal inference	Fetsch et al. (2009); Fetsch et al. (2011)
Conflict-dependent integration	Cross-modal attraction decreases as visual-vestibular disparity increases	Consistent with a transition from integration toward segregation, but similar patterns can arise from nonlinear weighting, robust integration, or switching	de Winkel et al. (2015, 2017); Rodriguez and Crane (2019)
Source-dependent integration	Visual influence changes when visual motion is attributed to self-motion versus independently moving objects	Provides stronger evidence that observers consider the likely source of visual motion	Dokka et al. (2019)
Model-based causal-inference evidence	Causal-inference models account for disparity-dependent changes in cue influence and can outperform forced-fusion models	Provides stronger support for causal inference, although conclusions depend on the alternative models tested and model identifiability	de Winkel et al. (2017); Acerbi et al. (2018)
Explicit and implicit causal judgments	Explicit unity judgments and implicit heading estimates vary with cue disparity and provide complementary constraints	Joint analysis better constrains causal inference and decision strategy, but does not establish a universal decision rule	Acerbi et al. (2018)
Experience-dependent recalibration	Previous visual-vestibular relationships alter subsequent unisensory and multisensory estimates	Demonstrates adaptive learning, but does not identify whether experience changes the common-cause prior, sensory estimates, uncertainty, or decision criteria	Zaidel et al. (2011); Zaidel et al. (2013)
Individual and strategy-level variability	Observers may differ in the extent and form of integration under cue conflict	Highlights that graded group-level behavior does not necessarily imply model averaging within individual observers	de Winkel et al. (2017); Acerbi et al. (2018)

## 4 Neural evidence and candidate mechanisms

Neural studies have identified several processes that could contribute to visual-vestibular causal inference, including multisensory convergence, reliability-dependent weighting, sensitivity to cue conflict, and experience-dependent changes in sensory representations. These findings establish that self-motion signals are processed flexibly across a distributed neural network. However, most experiments have examined the sensory and computational components required for causal inference rather than whether the brain represents the inferred relationship between the cues. The central question is therefore not whether visual and vestibular interactions occur in the brain, but whether neural activity reflects whether the signals are inferred to share a source, beyond effects of heading, reliability, disparity, and behavioral response. Table 4 summarizes the main classes of neural evidence and their relevance to visual-vestibular causal inference.

**Table 4:** Neural evidence relevant to visual-vestibular causal inference.

Evidence category	Regions	Main neural finding	Relevance to BCI	Representative studies
Multisensory convergence and heading coding	MSTd, VIP, VPS, PIVC, FEF_sem_	Visual and vestibular self-motion signals converge and encode heading	Establishes sensory representations required for integration, but provides no direct evidence for causal inference	Gu et al. (2006); Gu et al. (2008); Chen et al. (2010, 2011b,c, 2013); Gu et al. (2016)
Reliability-dependent weighting	MSTd	Neural cue contributions change with sensory reliability and relate to behavioral weights	Supports reliability-dependent integration, but not common-cause probability	Gu et al. (2008); Fetsch et al. (2011)
Conflict-dependent interactions	MSTd, VIP, VPS	Neural responses vary with cue congruence and disparity	Provides signals relevant to integration and segregation, but may arise from nonlinear interactions	Morgan et al. (2008); Chen et al. (2011c); Zhao et al. (2023)
Experience-dependent recalibration	MSTd, VIP, PIVC	Neural representations change following cue conflict or feedback	Shows history dependence, but does not identify which causal or sensory quantity is updated	Zaidel et al. (2021); Zeng et al. (2023)
Decision and report signals	VIP, FEF, PIVC	Activity correlates with heading choice or perceptual report	May reflect task-dependent readout, decision formation, or motor preparation	Ding and Gold (2012); Zaidel et al. (2017); Chen et al. (2021); Zeng et al. (2026)
Neural signals related to causal inference outside visual-vestibular self-motion	Premotor and parietal cortex	Trial-by-trial activity reflects conflict or the tendency to treat signals as belonging together	Provides stronger evidence for neural source inference, but not directly in visual-vestibular self-motion	Fang et al. (2019); Qi et al. (2022)

### 4.1 Distributed cortical network for multisensory self-motion

Visual-vestibular self-motion perception is supported by a distributed network of cortical and subcortical areas rather than by a single locus. Key cortical regions include the dorsal medial superior temporal area (MSTd) (Duffy and Wurtz, 1991; Lappe et al., 1996; Duffy, 1998; Bremmer et al., 1999; Page and Duffy, 2003; Gu et al., 2006), the ventral intraparietal area (VIP) (Schaafsma and Duysens, 1996; Duhamel et al., 1998; Bremmer et al., 2002a,b; Schlack et al., 2002; Zhang and Britten, 2010; Chen et al., 2011c), and the visual posterior sylvian cortex (VPS) (Chen et al., 2011b), parieto-insular vestibular area (PIVC) (Chen et al., 2010). Although these areas differ in their relative visual and vestibular weighting, together they form a distributed network for representing self-motion.

MSTd contains neurons selective for optic-flow patterns associated with translational and rotational self-motion and also receives vestibular heading signals, allowing the area to represent heading from either modality or their combination (Lappe et al., 1996; Duffy, 1998; Bremmer et al., 1999; Gu et al., 2006; Gu et al., 2008). VPS and PIVC are more strongly driven by inertial motion and are often described as vestibular-dominant components of the network (Chen et al., 2010, 2011b,c). VIP also carries visual and vestibular self-motion signals, but its activity is strongly modulated by task demands and behavioral choice (Schaafsma and Duysens, 1996; Zhang and Britten, 2010; Chen et al., 2011c; Zaidel et al., 2017; Zaidel et al., 2021; Zeng et al., 2023; Zeng et al., 2026).

This regional diversity is unlikely to reflect a simple progression from a visual area to a vestibular area and then to a dedicated causal-inference area. Instead, the network contains partially overlapping representations of heading, sensory uncertainty, cue conflict, and behavioral choice. MSTd, VIP, VPS and PIVC therefore provide different neural constraints on visual-vestibular inference, but none has yet been established as the site at which common versus separate causes are explicitly represented. Figure 3 summarizes the candidate neural regions and the computational quantities for which current evidence is direct, indirect, or still lacking.

**Figure 3:**
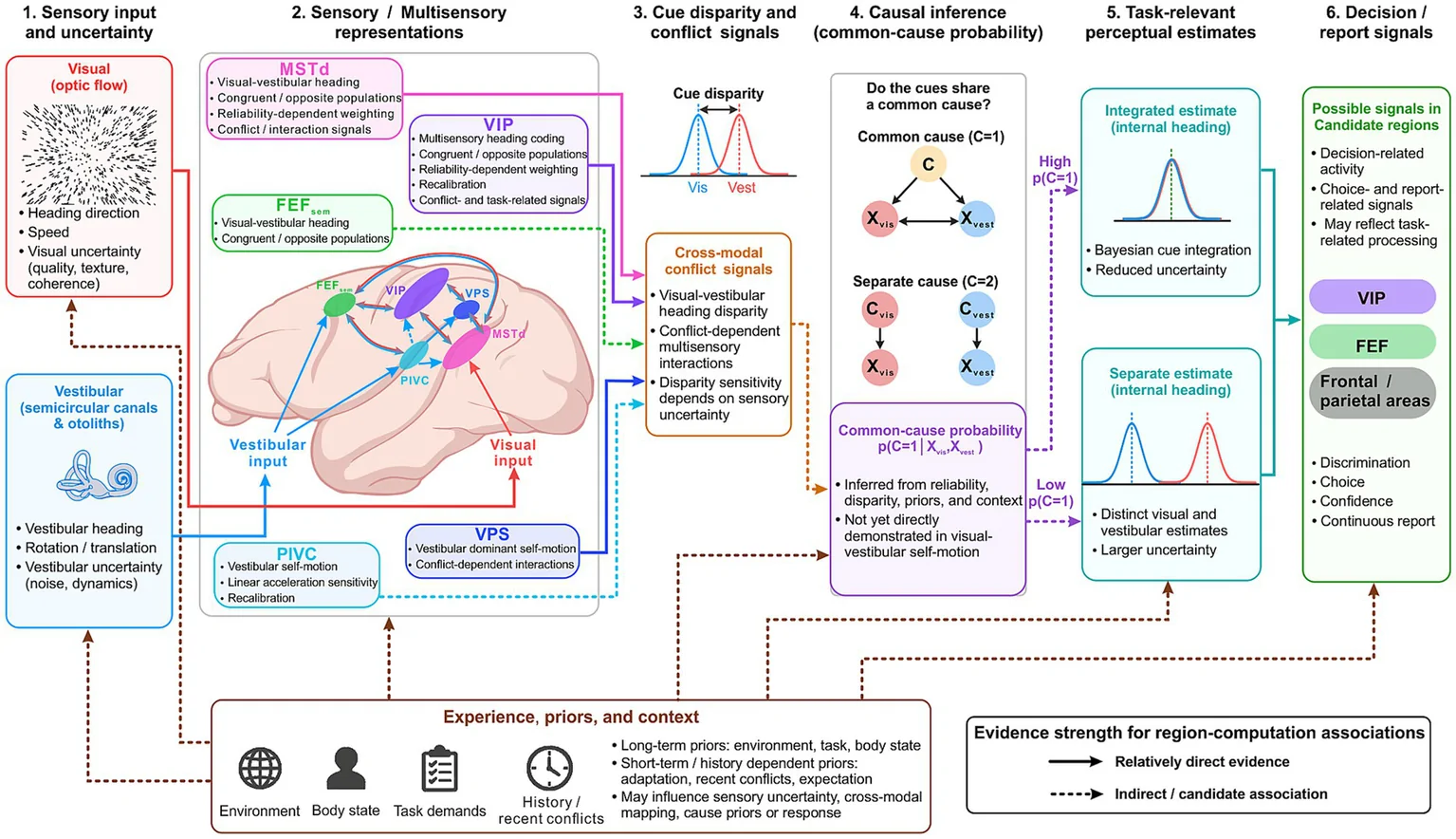
Candidate neural substrates of computational processes relevant to visual-vestibular causal inference in self-motion perception. Visual and vestibular inputs provide information about self-motion and sensory uncertainty (1). These signals are represented across a distributed network of candidate regions (2), including MSTd, VIP, FEF_sem_, PIVC, and VPS, which differ in their sensory dominance and reported sensitivity to heading, cue reliability, multisensory interactions, recalibration, and task-related signals. Visual-vestibular disparity and conflict-dependent interactions provide information about the correspondence between the two sensory signals (3). In a Bayesian causal-inference framework, this information, together with sensory uncertainty, prior expectations, and context, contributes to the inferred probability that the cues share a common cause, *p*(*C* = 1 | *x*_vis_, *x*_vest_) (4). A direct neural representation of this common-cause probability has not yet been demonstrated in visual-vestibular self-motion. Depending on the inferred causal relationship, visual and vestibular information may contribute to an integrated estimate or remain as separate estimates (5), which can subsequently contribute to perceptual decisions and behavioral reports (6). Experience, prior expectations, and context may influence multiple stages of this process, including sensory uncertainty, cross-modal mapping, causal prior, and response processes. Solid arrows indicate relatively direct evidence from visual-vestibular self-motion studies, whereas dashed arrows indicate indirect or candidate associations. MSTd, the dorsal medial superior temporal area; VIP, the ventral intraparietal area; VPS, the visual posterior sylvian area; FEF_sem_, the smooth eye movement area of the frontal eye field; PIVC, the parieto-insular vestibular cortex.

### 4.2 Reliability-dependent weighting: a neural basis for flexible integration

A central property of the self-motion network is that neural representations change with the reliability of the available sensory signals. When optic-flow reliability is reduced, behavioral heading judgments shift toward vestibular information; when visual motion is more precise, visual signals exert greater influence. Corresponding changes are found in MSTd, where population responses and neural estimates of cue weighting vary with visual reliability and are related to the weights expressed in the animal’s behavior (Gu et al., 2008; Fetsch et al., 2011). These findings provide a neural basis for reliability-weighted visual-vestibular integration. Rather than combining the two inputs with fixed coefficients, multisensory populations adjust their contribution as sensory uncertainty changes. Such flexibility is consistent with Bayesian estimation and contributes to improved heading precision when the cues provide compatible information.

Importantly, however, reliability-dependent neural weighting should be distinguished from neural evidence for causal inference. The same pattern is expected from optimal integration models that assume from the outset that the cues describe a common self-motion event. Within a causal-inference model, sensory uncertainty also affects how much evidence a given disparity provides against a common source, but current MSTd results mainly establish reliability-dependent cue weighting (Gu et al., 2008; Fetsch et al., 2011). They do not directly test whether neural activity represents common-cause probability.

Thus, sensory populations may supply uncertainty-dependent evidence that is subsequently combined with cue disparity and prior expectations elsewhere in the network. Alternatively, reliability and cue-conflict information may be combined within the same distributed population. Present data do not distinguish clearly between these possibilities. Distinguishing these possibilities requires analyses that relate neural activity directly to model-derived common-cause probability rather than to cue weights alone.

### 4.3 Cue conflict and cross-modal interactions

Neural interactions also depend on the correspondence between visual and vestibular heading signals. Across MSTd, VIP, VPS, FEFsem, and related multisensory areas, neurons vary substantially in the alignment of their visual and vestibular preferences (Gu et al., 2006; Chen et al., 2010, 2011b,c; Gu et al., 2016; Zhao et al., 2021). Congruent neurons have similar preferred headings for the two modalities, whereas opposite neurons prefer substantially different directions. This organization provides the network with information about both similarities and differences between visual and vestibular heading signals.

In MSTd, congruent neurons generally contribute most strongly to improved heading discrimination when visual and vestibular cues are aligned (Gu et al., 2008; Morgan et al., 2008). The tuning of opposite neurons makes them sensitive to differences between visual and vestibular heading signals (Gu et al., 2008; Morgan et al., 2008), although their role in perception is less certain. They are therefore plausible contributors to cue-disparity signals, but it has not been shown that their activity directly controls whether the cues are integrated or segregated. Their functional role may also extend beyond causal inference.

Combined-cue responses further differ across cortical areas. Responses in MSTd are commonly subadditive, meaning that the combined response is smaller than the sum of the two unimodal responses, and depend on both unimodal tuning and the alignment of the two inputs (Gu et al., 2008; Morgan et al., 2008). In vestibular-dominant VPS, visual stimulation can suppress vestibular responses, with the strength of the interaction varying with visual-vestibular disparity (Zhao et al., 2023). VIP contains more balanced visual and vestibular representations and shows substantial modulation of response gain under combined stimulation (Chen et al., 2011c; Zaidel et al., 2017). These differences indicate that cue conflict is not represented by a uniform neural operation throughout the self-motion network.

Conflict-dependent enhancement and suppression are relevant to causal inference, because they alter the effective contribution of each modality as their correspondence changes. However, these effects are not specific to causal inference. Divisive normalization, nonlinear summation, and cross-modal suppression can all produce disparity-dependent changes in multisensory responses without first computing a neural estimate of how likely the visual and vestibular signals are to arise from the same source (Ohshiro et al., 2011, 2017; Zhao et al., 2023). The neural response to cue conflict therefore constrains possible implementations of integration and segregation, but it does not by itself reveal how the brain attributes the signals to their sources.

### 4.4 Experience-dependent recalibration and task-related signals

Visual-vestibular representations can change after sustained cue conflict or feedback-guided learning (Zaidel et al., 2021; Zeng et al., 2023). These changes differ across cortical areas. Following prolonged visual-vestibular discrepancy, visual and vestibular tuning in MSTd shifted in the same directions as their respective perceptual recalibrations, whereas vestibular tuning in PIVC followed the vestibular perceptual shift. In VIP, by contrast, both visual and vestibular tuning shifted with the vestibular perceptual recalibration, such that the visual neuronal shift was opposite to the visual perceptual shift (Zeng et al., 2023). These differences indicate that recalibration is not implemented as a uniform adjustment across the self-motion network.

Feedback-guided (supervised) recalibration, in which external feedback about heading is used to adjust subsequent multisensory estimates, reveals a further distinction between sensory and task-related representations. Recalibration-related activity was evident in VIP but was weak or absent in MSTd during supervised calibration (Zaidel et al., 2021). This difference is consistent with a greater involvement of VIP in linking multisensory estimates to behavioral reports, whereas MSTd may be more closely related to the sensory representation of heading. However, it does not imply that VIP uniquely implements causal inference, because feedback, choice, error signals, and changes in perceptual readout can all influence its responses.

Recalibration is relevant to causal inference because previous visual-vestibular relationships can alter the interpretation of subsequent signals. Neural changes after exposure could reflect updated sensory estimates, modified cue weights, altered cross-modal mappings, or changes in expectations about whether the signals share a source. Together, the neural findings demonstrate experience-dependent changes in multisensory cortical representations (Zaidel et al., 2021; Zeng et al., 2023), but do not identify which sensory or causal quantity is being changed.

Task-related modulation—neural activity associated with an animal’s choice or report beyond that explained by the sensory stimulus—introduces a similar interpretive problem. Choice-related activity has been reported in areas involved in visual-vestibular heading processing, including VIP and PIVC (Zaidel et al., 2017; Chen et al., 2021), whereas downstream decision- and report-related signals are also prominent in frontal oculomotor areas such as the FEF (Ding and Gold, 2012; Gu et al., 2016). Such activity may contribute to the readout of multisensory evidence, but decision-related correlations do not by themselves establish whether the signal reflects sensory inference, decision formation, feedback, or motor preparation (Katz et al., 2016; Zaidel et al., 2017). Separating these possibilities will require experiments in which the same visual-vestibular evidence supports different reports, or in which explicit source judgments and implicit heading estimates are measured within a common paradigm.

### 4.5 Population and circuit mechanisms for causal inference

The neural findings reviewed above identify several operations that could support visual-vestibular causal inference, including the representation of heading and sensory uncertainty, reliability-dependent cue weighting, sensitivity to cross-modal disparity, and task-dependent readout (Gu et al., 2008; Chen et al., 2010, 2011b,c; Fetsch et al., 2011; Gu et al., 2016; Zaidel et al., 2021; Zeng et al., 2023; Zhao et al., 2023). What remains uncertain is whether these signals are combined into a neural estimate of common-cause probability.

Probabilistic population coding, in which the activity of a neural population jointly represents a sensory estimate and its uncertainty, provides one possible framework for this process. Neural populations can therefore supply the information required for reliability-weighted integration and probabilistic readout (Ma et al., 2006; Beck et al., 2008; Ma and Rahmati, 2013). Congruent and opposite neurons may provide complementary information about shared sensory estimates and cross-modal conflict, thereby supporting integration- and segregation-like computations (Zhang et al., 2019). However, these population mechanisms do not necessarily require the brain to represent common-cause probability.

Several network models illustrate how these computations might be implemented neurally (Cuppini et al., 2017; Terada and Toyoizumi, 2024; Ponzi and Suzuki, 2025). A biologically inspired audiovisual network containing interacting unisensory and multisensory populations reproduced sensory biases, unity judgments, and changes between integration and segregation consistent with causal inference (Cuppini et al., 2017). Chaotic recurrent dynamics provide another possible mechanism: neural sampling can support Bayesian cue integration and represent uncertainty, although this model did not explicitly distinguish common and separate causes (Terada and Toyoizumi, 2024). A more recent chaotic-network model generated common-cause probabilities, integrated and segregated perceptual estimates, and history-dependent fluctuations consistent with BCI (Ponzi and Suzuki, 2025). Together, these models provide neurally plausible computational principles, but none has yet been tested directly in visual-vestibular self-motion circuits.

A feedforward network trained on visual-vestibular motion estimation likewise developed congruent and opposite units whose relative activity supported motion estimation and judgments about whether sensory signals shared a source (Rideaux et al., 2021). This work provides an important computational account of how causal structure could emerge from visual-vestibular representations, but it does not constitute evidence that recorded neural populations implement the same computation. Similarly, human fMRI, EEG, and MEG studies in other multisensory domains suggest that modality-specific estimates, integrated estimates, and behavior related to causal inference may be expressed across different cortical levels and time windows (Rohe and Noppeney, 2015; Cao et al., 2019; Rohe et al., 2019). Primate electrophysiology in visuoproprioceptive conflict tasks further shows that premotor and parietal activity can track conflict magnitude and the trial-by-trial tendency to treat the signals as belonging together (Fang et al., 2019; Qi et al., 2022). These findings offer possible principles for visual-vestibular processing, but they are not direct evidence from self-motion paradigms.

Overall, current visual-vestibular evidence therefore supports a distributed network that represents the sensory estimates, uncertainty, cue relationships, and task-related signals required for flexible multisensory behavior. It does not yet establish whether common-cause probability is represented explicitly or whether integration and segregation emerge from nonlinear population interactions and later decision processes. In particular, no visual-vestibular self-motion study has yet linked trial-by-trial neural activity to a model-derived estimate of common-cause probability while separating it from heading, sensory reliability, cue disparity, choice, and motor report.

## 5 Open questions and future directions

Behavioral and neural studies have established that visual-vestibular interactions vary with cue reliability, disparity, task demands, and previous experience. The remaining challenge is to determine when this flexibility reflects inference about whether sensory signals share a source rather than changes in sensory reliability, cue weighting, or decision strategy. Future studies should manipulate source attribution directly and compare Bayesian causal-inference models with plausible alternatives, including forced fusion, cue switching, robust integration, and probability-matching strategies. Combining explicit source judgments with implicit perceptual measures and individual- or trial-level analyses may provide stronger constraints on the computations underlying integration and segregation (Acerbi et al., 2018; Cao et al., 2019; Dokka et al., 2019).

A closer link between computational models and neural activity is also needed. MSTd, VIP, VPS/PIVC, and associated regions encode heading, reliability, cue conflict, recalibration, and behavioral choice, but it remains unclear whether any part of this network represents common-cause probability itself. Relating trial-by-trial population activity to model-derived source estimates may help distinguish neural signals related to causal inference from sensory and decision variables (Qi et al., 2022). Combining such analyses with perturbations of candidate regions could further test whether these signals contribute selectively to decisions about integration versus segregation, rather than to heading estimation or motor report. Neurally plausible models and population-coding models may provide useful hypotheses for how such computations could emerge from distributed circuits, but these predictions remain to be tested in visual-vestibular self-motion.

Finally, current models are based largely on passive motion and a common- versus separate-cause distinction. Natural self-motion also involves proprioception, motor commands, eye movements, and multiple moving elements in the environment. Extending causal-inference models to active and more complex environments will therefore be essential for understanding self-motion outside controlled laboratory conditions. These questions may also be important for virtual environments and vestibular disorders, where unstable perception could arise from changes in sensory precision, calibration, cue weighting, or source attribution. Distinguishing among these mechanisms will be necessary before causal-inference models can guide practical applications.

## 6 Concluding remarks

Visual and vestibular signals are combined flexibly during self-motion perception. Their relative influence changes with sensory reliability, cue disparity, task demands, and experience, providing strong evidence against fixed weighting and obligatory fusion. Bayesian causal inference offers a useful framework for explaining how sensory uncertainty and beliefs about whether the cues share a common cause jointly influence their combination. However, the strength of evidence varies across phenomena: behavior that depends on the likely source of visual motion and quantitative comparison with plausible alternative models provide stronger support for causal inference, whereas reliability-dependent weighting, disparity-dependent cue attraction, and recalibration are compatible with, but not specific to, this framework.

Neural studies have identified a distributed visual-vestibular network encoding heading, sensory uncertainty, cue conflict, recalibration, and behavioral choice. These findings establish many of the neural processes that could support causal inference, but whether the brain explicitly represents common-cause probability remains unresolved. Future progress will require tighter integration of computational modeling, population-level neural measurements, and causal perturbation, particularly during active and naturalistic self-motion. Such approaches may ultimately determine whether visual-vestibular causal inference involves an explicit neural estimate of whether the cues share a common cause, or instead emerges from distributed interactions among sensory, uncertainty, and decision-related signals.
